# Stability of amino acids and related amines in human serum under different preprocessing and pre-storage conditions based on iTRAQ^®^-LC-MS/MS

**DOI:** 10.1242/bio.055020

**Published:** 2021-02-26

**Authors:** Zhuoling An, Chen Shi, Pengfei Li, Lihong Liu

**Affiliations:** Pharmacy Department of Beijing Chao-Yang Hospital, Capital Medical University, Beijing, 100020, PR China

**Keywords:** Amino acid and related amines, Stability, Serum, LC-MS/MS, iTRAQ

## Abstract

Amino acid analysis or metabonomics requires large-scale sample collection, which makes sample storage a critical consideration. However, functional amino acids are often neglected in metabolite stability studies because of the difficulty in detecting and accurately quantifying them with most analysis methods. Here, we investigated the stability of amino acids and related amines in human serum following different preprocessing and pre-storage procedures. Serum samples were collected and subjected to three storage conditions; cold storage (4°C), room temperature storage (22°C), and freezing (−80°C). The concentration of amino acids and related amines were quantified using iTRAQ^®^-LC-MS/MS with isobaric tagging reagents. Approximately 54.84%, 58.06%, and 48.39% of detectable and target analytes were altered at the 4°C condition, 22°C condition, and when subjected to freeze-thaw cycles, respectively. Some amino acids which are unstable and relatively stable were found. Our study provides detailed amino acid profiles in human serum and suggests pre-treatment measures that could be taken to improve stability.

## Abstract (precis)

**Summary:** We investigated the stability of amino acids in serum samples that underwent prolonged storage at 4°C and 22°C, and repeated freeze-thaw cycles at −80°C using stable isotope iTRAQ labeling and liquid chromatography tandem mass spectrometry.

## INTRODUCTION

The detection and quantification of free amino acids is routinely applied in clinical laboratories for the diagnosis of inborn errors of metabolism (Phipps et al., 2019). Amino acid synthesis defects accompany many clinical symptoms including the central nervous system and mental disability for children, skin disorders such as cutis laxa in defects of proline synthesis, collodion-like skin and ichthyosis in serine deficiency, and necrolytic erythema in glutamine deficiency (de Koning, 2017). Furthermore, free amino acids have been implicated in having a role in a number of diseases such as cardiovascular diseases (Poggiogalle et al., 2019; Nie et al., 2018; Larsson et al., 2015), insulin resistance and type 2 diabetes (Yoon, 2016; Gar et al., 2018), renal diseases (Xia et al., 2018), hepatic disorders (Korenaga et al., 2015), and cancer (Li and Zhang, 2016; Yang and Vousden, 2016).

Increasing interest in amino acids related to diseases has prompted the need for verifying their stability and sensitivity under different sample processing conditions. Existing studies have focused on long-term and short-term stability during sample storage. It was found that the storage of plasma samples at −80°C for up to 5 years leads to changes in the concentration of amino acids, acylcarnitines, glycerophospholipids, sphingomyelins, and hexoses (Haid et al., 2018). Increased levels of amino acids during storage were also observed in other long-term stability studies (Pinto et al., 2014; Trabi et al., 2013).

The human plasma metabolome was found to be adequately stable during long-term storage at −80°C for up to seven years, however, cysteine and cystine were seen to decrease over longer storage times (Wagner-Golbs et al., 2019). Amino acids in serum were found to have reacted during 29 years of long-term storage at −25°C with methionine being transformed to methionine sulfoxide (Hustad et al., 2012).

After one freeze-thaw cycle, 18 free amino acids in urine samples were found to be stable for 72 h at 4°C, but for no more than 4 weeks at −80°C (Joyce et al., 2016). When delayed freezing was used the opposite result was found (La Frano et al., 2018). The concentration of arginine, glycine, ornithine, phenylalanine, serine, and isoleucine increased significantly during pre-storage handling at room temperature while glutamine decreased slightly at room temperature or on wet ice for 36 h (Anton et al., 2015). Stability in blood and plasma were also different because the platelets became activated and their metabolism was affected by the low temperature (Kamlage et al., 2014).

Amino acids in blood were significantly affected by pre-analytical short-term storage with changes at room temperature observed after 2 h, and on wet ice after 6 h, whereas changes in the amino acids in plasma for 16 h at room temperature were not observed (Kamlage et al., 2014). Changes in the concentration of glutamate in both of blood and plasma were observed, while taurine changed only in the blood samples through the same procedure (Kamlage et al., 2014). Some amino acids and biogenic amines could become unstable within 3 h on cool packs. Isoleucine, tryptophan, and valine evidently decreased when undergoing two freeze-thaw cycles (Breier et al., 2014). Nine amino acids were stable for up to 24 h in plasma at 37°C, among which some amino acids changed statistically when the plasma specimens were placed at 4°C for 24 h (Yang et al., 2013). The amino acids exhibited significant time-of-day variations (Ang et al., 2012). Alanine and other metabolites changed after four or five freeze-thaw cycles at room temperature (Pinto et al., 2014; Fliniaux et al., 2011).

The stability of amino acids and related amines in human serum varies under different preprocessing and pre-storage conditions, understanding this is critical to medical researchers because of the large number of serum/plasma samples collected for amino acids analysis. However, a large number of amino acids may have been missed in previous studies because of the limitations the approaches used and the scope of study. Because of the lack of comprehensive research on amino acid stability, there is no comparison of multiple amino acids in the same dimension in one study, which leads to inconsistent and potentially contradictory conclusions.

The most common amino acid analysis has been cation-exchange and reversed phase liquid chromatography coupled to pre-column or post-column derivatized UV optical detection. However, co-eluting substances cannot be distinguished and quantified by these conventional approaches because of the lack of analyte specificity and selectivity. We have developed a novel approach using stable isotope iTRAQ labeling and liquid chromatography tandem mass spectrometry to achieve comprehensive profiling and quantification of 42 amino acids and related amines (An et al., 2020). Compared with typical MS-based methods, the iTRAQ^®^-LC-MS/MS has internal standards available for all the analytes (An et al., 2020). Our study aims to estimate the stability of amino acids and related amines in serum samples after exposure to adverse storage temperatures and freeze-thawing cycles by using iTRAQ^®^-LC-MS/MS. The delineation of amino acid stability is based on a comprehensive profiling and quantification approach of amino acids.

## RESULTS AND DISCUSSION

### Quantification of amino acids and related amines by iTRAQ-based profiling

In our previous study, norvaline was added to the reaction system to investigate the derivatization efficiency of amino acids and related amines (An et al., 2020). The results showed more than 80% norvaline could be derivatized by the iTRAQ reagent. In this study, 31 labeled amino acids and related compounds were separated and quantified with excellent peak shapes. The MRM ion chromatograms corresponding to the amino acids and their isotopic internal standards were extracted from the data. Integration and calculation were adjusted to quantify amino acid levels.

**Fig. 1.**
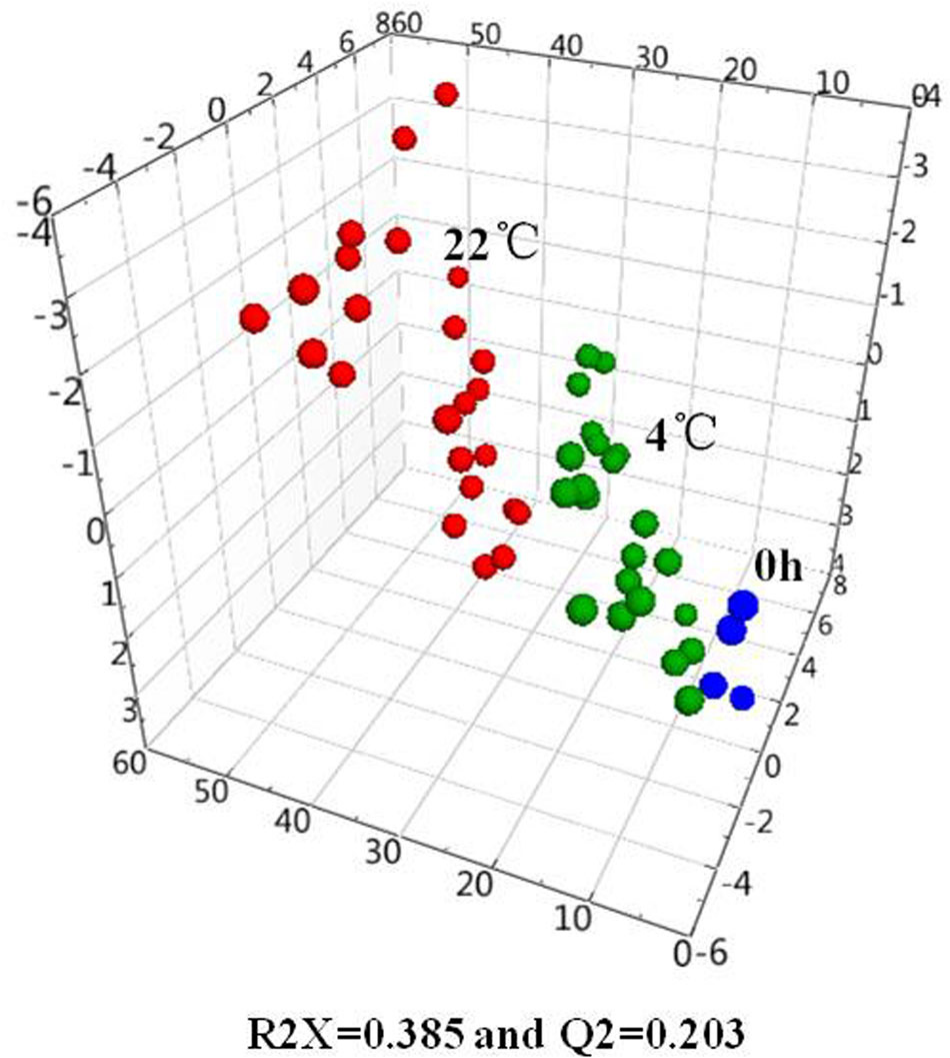
**PCA score plot of the concentration of amino acids in human serum deposited at 4°C (green spheres), 22°C (red spheres) and samples detected immediately after processing (blue spheres).**

**Fig. 2.**
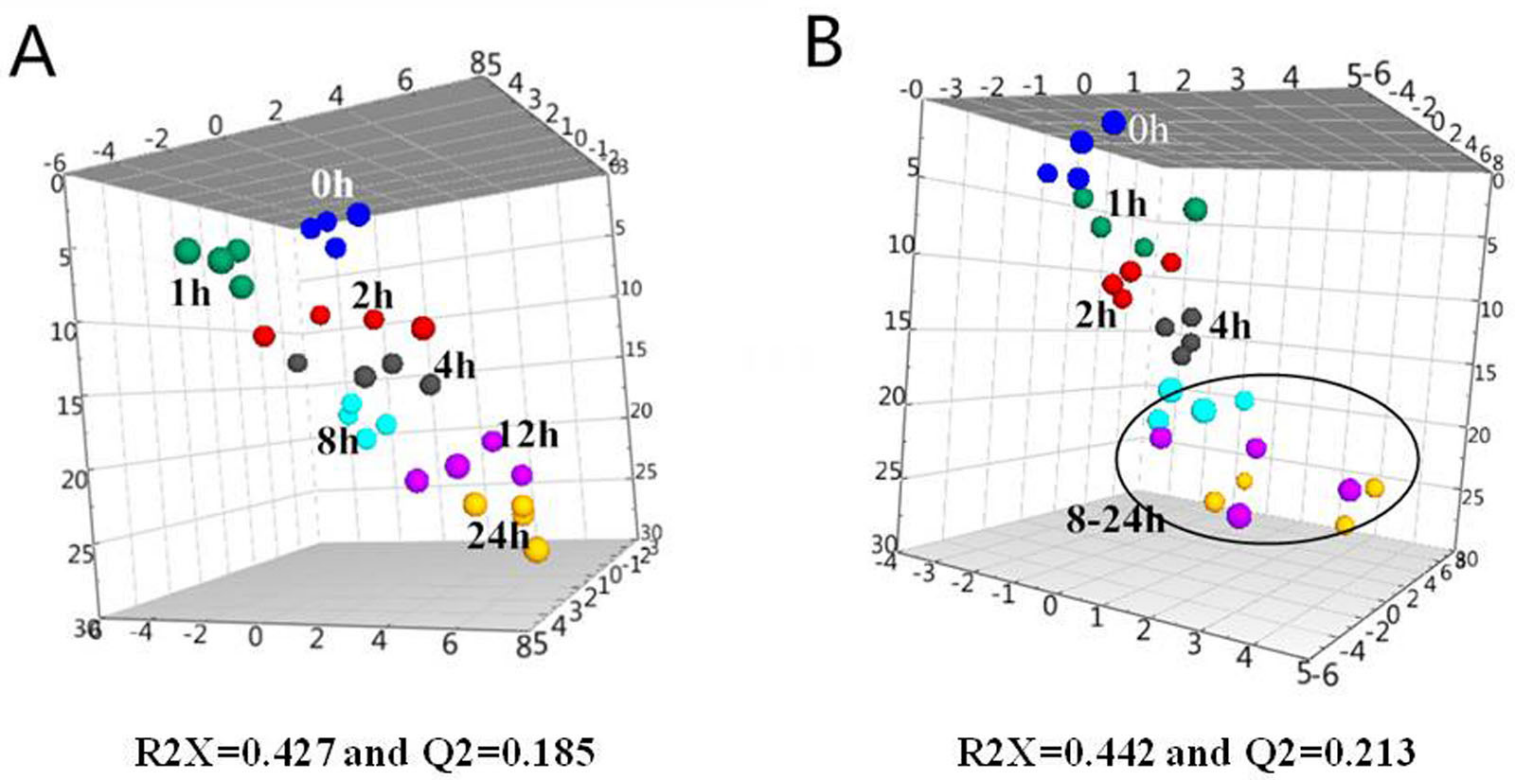
**PCA score plot of the serum samples deposited at 4°C (A) and 22°C (B) from 0 h to 24 h (blue spheres, 0 h; green spheres, 1 h; red spheres, 2 h; grey spheres, 4 h; aqua spheres, 8 h; magenta spheres, 12 h; yellow spheres, 24 h).**

Principal component analysis (PCA) could provide clustering information in each group and possible metabolic profile changes. The concentrations were assigned as variables while the pre-storage handing conditions and freeze-thaw cycles were set as factors for the multivariate data analysis. SIMCA-P 13.0 (VersionAB, Umeå, Sweden) was employed to visually investigate the clustering of amino acids measured in serum at different pre-processing conditions. Fig. 1 shows the three-dimensional PCA score plot of serum samples stored at 4°C and 22°C with different storage times as well as samples detected immediately after processing. The model statistics indicate a low degree of fit (R2X=0.385) and low predictability (Q2=0.203). Multivariate data analysis by PCA did not show clear separation of the three groups. However, the serum samples stored at 4°C, 22°C, and the samples detected immediately after processing tended to aggregate together. This indicated that there were indeed differences among the serum samples stored at different conditions and the differences were gradually changing. The samples stored at 22°C were relatively scattered, and more variability was found in samples analyzed immediately after processing. We also performed a univariate analysis comparing the serum samples at the different storage times. Fig. 2 shows the three-dimensional PCA score plot of serum samples for different storages times (0, 1, 2, 4, 8, 12 and 24 h) at 4°C (A) and 22°C (B). The values for the two multivariate analyses at 4°C and 22°C were R2X=0.427, Q2=0.185, and R2X=0.442, Q2=0.213, respectively. Although the R2X and Q2 values from the PCA model were low, the sample aggregation at the same times and temperatures is good. The serum samples at 4°C from 0 to 24 h are scattered and distributed with increased storage time, the samples gathered at the same pre-storage times gradually deviated from immediately detected samples. When stored at 22°C, the serum samples are scattered and distributed with storage time. After 8 h, they deviate from the sample group with rapid detection significantly. Taken together, the variability stems from variations in amino acid content as a function of time and temperature. It shows that temperature and storage time have an impact on the composition of amino acids in serum.

### Stability of serum amino acids at 4°C

**Fig. 3.**
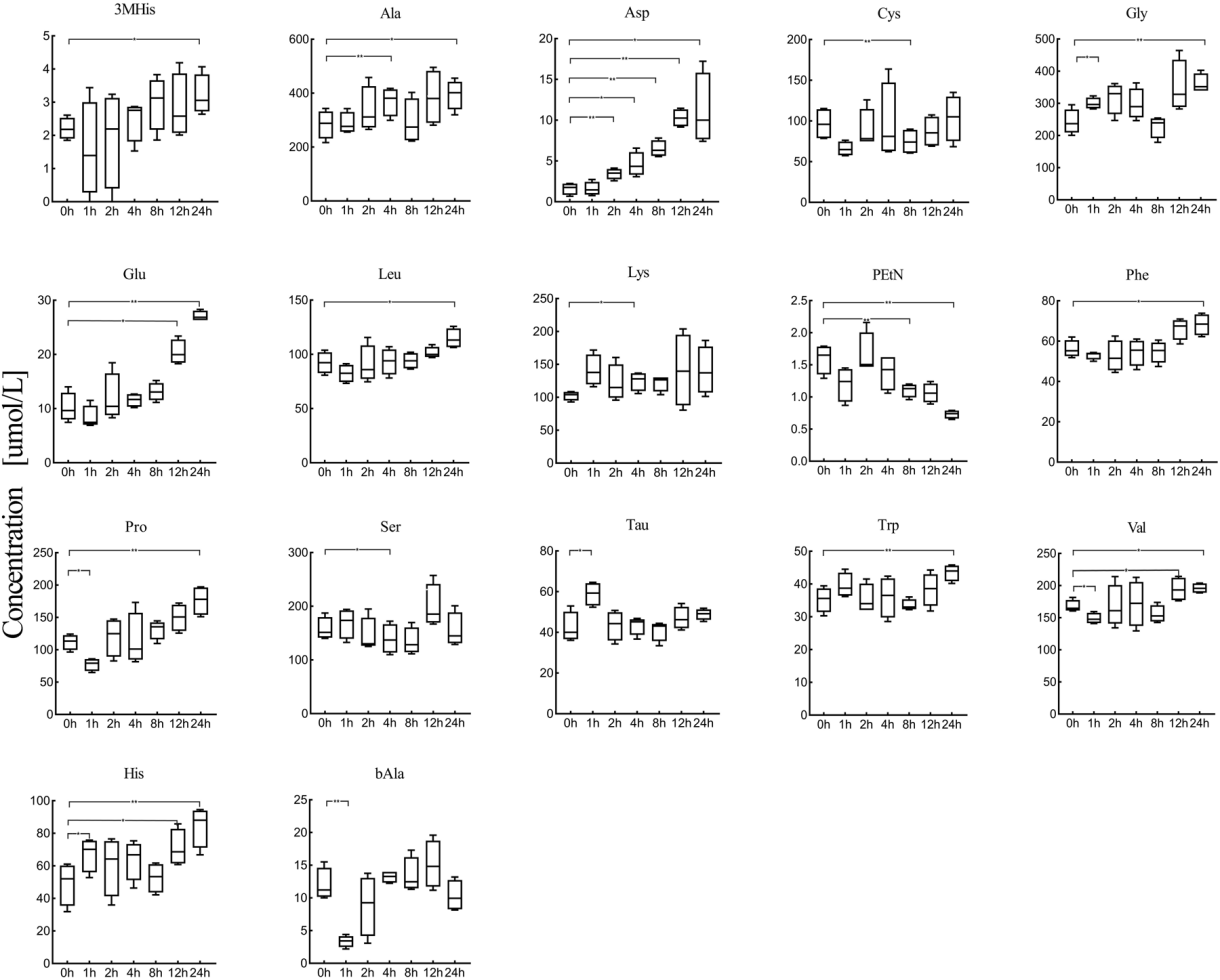
**The changing trend of 17 amino acids in serum after serum specimens were incubated at 4°C for 24 h.** The amino acid quantitative results at different times were compared with the amino acid concentration at 0 h to identify the significance of change. (*P<0.05, **P<0.01).

17 amino acids in serum were significantly affected at 4°C across the different pre-processing periods (*P*<0.05) (Fig. 3). The concentration and change ratios of these amino acids changed significantly after the serum specimens were incubated at 4°C for 24 h (Table S1). 3-methylhistidine, alanine, aspartate, glycine, glutamate, leucine, phenylalanine, proline, tryptophan, valine and histidine increased significantly after 24 h. Taurine and lysine was increased to significantly higher levels within 1 h and 4 h, while cystine as well as phosphoethanolamine decreased after 8 h. *β*-alanine and serine decreased after 1 h and 4 h. Notably, aspartate remained relatively stable for 1 h and increased rapidly afterwards where an approximate 700-fold change was observed. Glutamate also increased continuously after 12 h with a change ratio of 266%.

### Stability of serum amino acids at 22°C

**Fig. 4.**
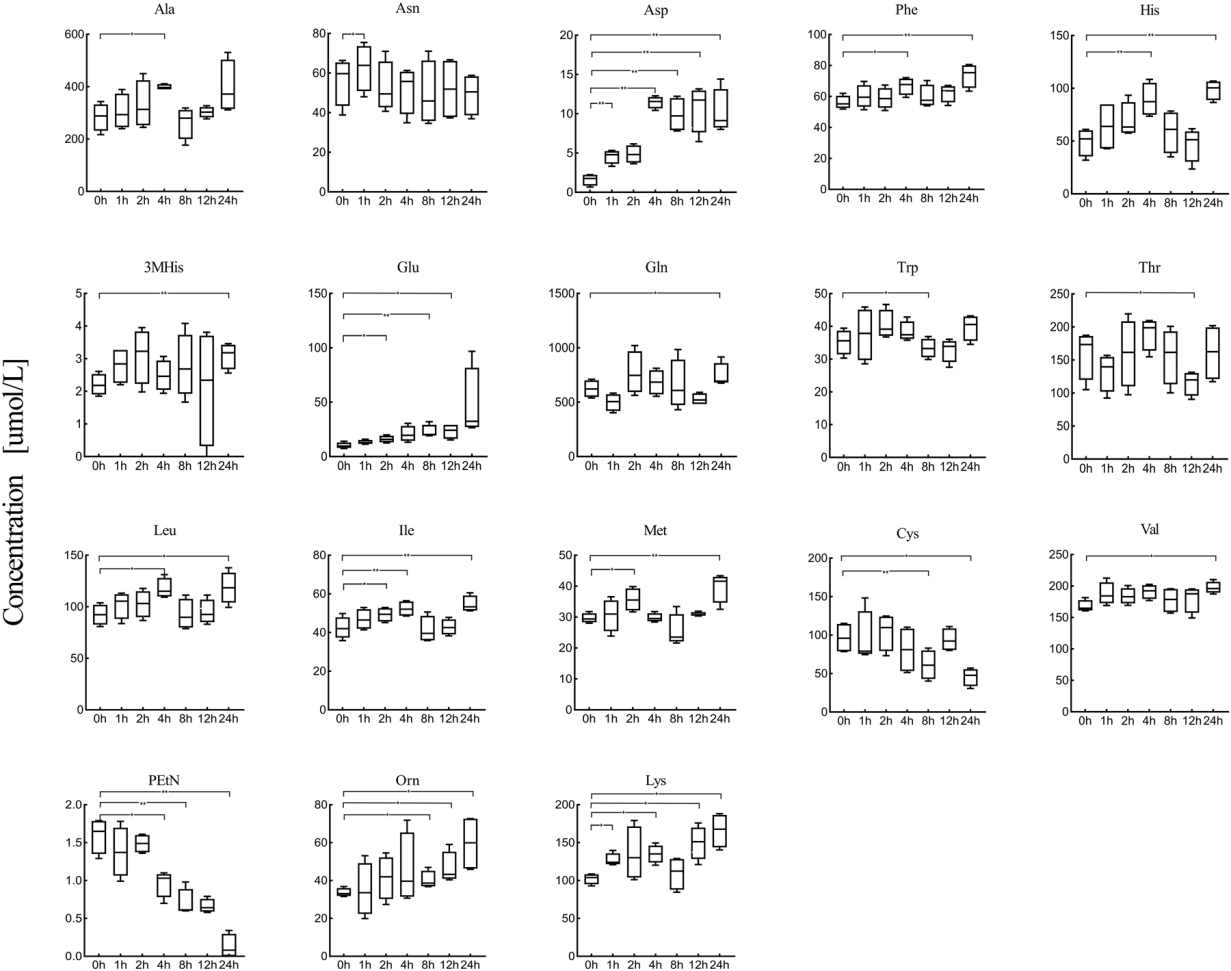
**The changing trend of 18 amino acids in serum after serum specimens incubated at 22°C for 24 h.** The amino acid quantitative results at different times were compared with the amino acid concentration at 0 h to identify the significance of change. (*P<0.05, **P<0.01).

The levels of 18 amino acids were significantly affected after 24 h of 22°C storage after serum sample preprocessing (*P*<0.05) (Fig. 4). Aspartate, phenylalanine, histidine, 3-methylhistidine, glutamine, isoleucine, leucine, methionine, valine, ornithine and lysine increased strikingly after 24 h (Table S2). A reduction of cystine and phosphoethanolamine occurred after 24 h of storage, while threonine and tryptophan decreased within 12 h. The levels of asparagine, glutamate and alanine were noticeably elevated after 1–4 h. Moreover, an approximate 300-fold change of aspartate occurred within 1 h and increased steadily thereafter. The change ratio of aspartate had reached almost 634% after 24 h. Glutamate continuously increased after 2 h of preprocessing and the change ratio was about 462%. Cystine and phosphoethanolamine decreased by approximately 16–47% compared with their initial concentration after 24 h of storage.

### Stability of serum amino acids after three freeze-thaw cycles at −80°C

**Fig. 5.**
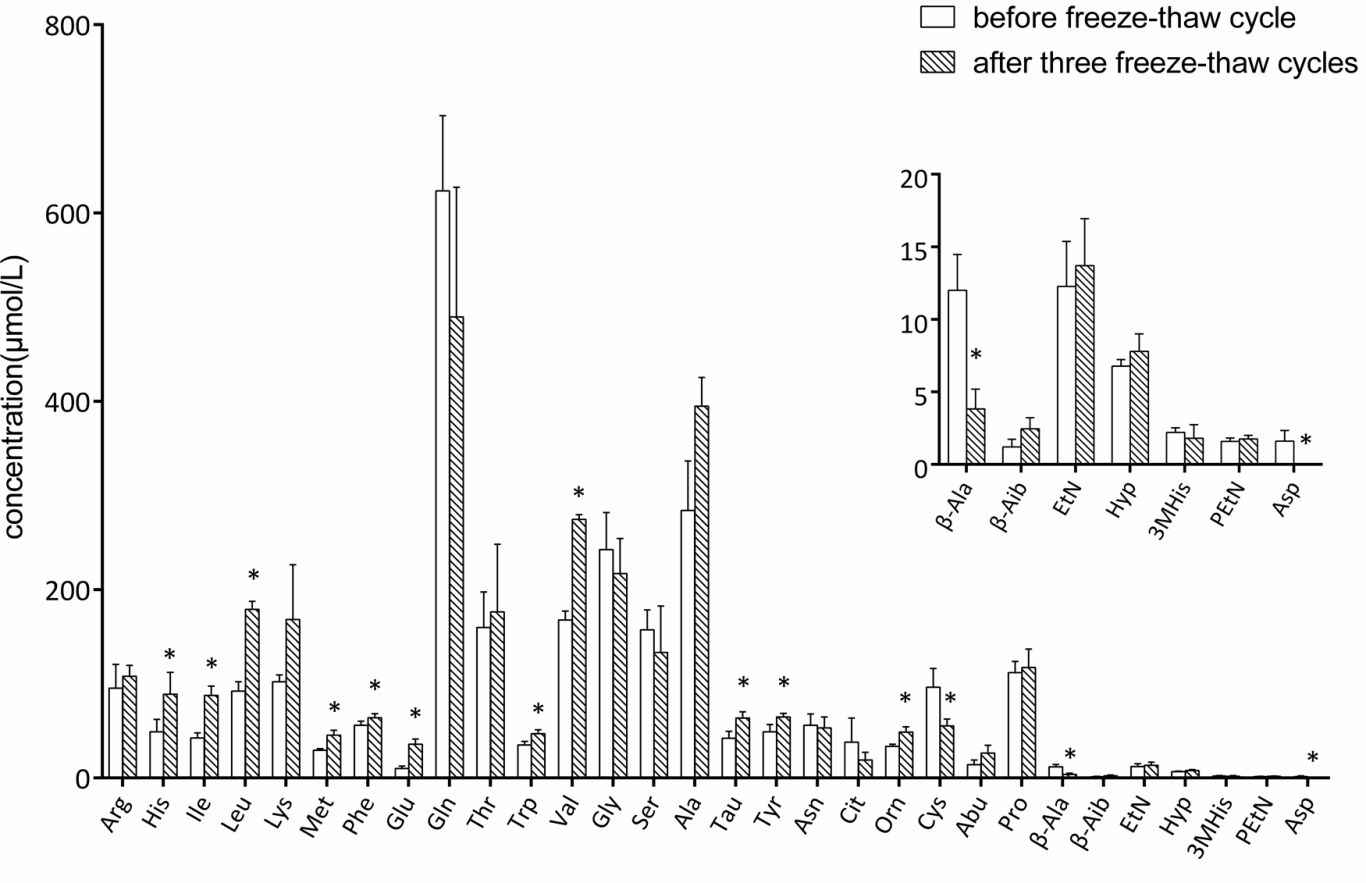
**Change of amino acids in serum after three freeze-thaw cycles and the concentrations of 14 amino acids in the serum compared with the samples without freeze-thaw process**. The freeze-thaw cycle samples were brought from −80°C, placed at 4°C for 1 h and then −80°C for 12 h. (*P*<0.05).

After the serum samples were frozen and thawed three times at −80°C, the concentrations of 11 amino acids (histidine, leucine, isoleucine, methionine, phenylalanine, glutamate, tryptophan, valine, taurine, tyrosine, and ornithine) in the serum increased strikingly compared with the samples without freeze-thaw treatment (*P*<0.05). Four amino acids including cystine, *β*-alanine, 1-methylhistidine, and aspartate reduced in concentration after the freeze-thaw cycle (*P*<0.05) (Fig. 5). The increased change ratio for these amino acids varied from 31.45% to 252.12% while that of the reduced change ratio was 42.57% to 100.00% (Table S3).

### Significant concentration changes in amino acids due to pre-storage handling

Our experiment with different pre-storage handling conditions showed pronounced changes of the detectable amino acids and related amines. Approximately 54.84%, 58.06%, and 48.39% of target analytes altered at 4°C, 22°C, and with freeze-thaw cycles during preprocessing, respectively. Seven amino acids (histidine, leucine, phenylalanine, tryptophan, valine, aspartate, and cystine), were more sensitive with evident modifications by multiple factors including different storage time and temperature as well as repeated freeze-thaw cycles. Conversely, arginine, tyrosine, citrulline, *α*-amino-N-butyric acid, *β*-aminoisobutyric acid, ethanolamine, and hydroxyproline were more stable and remained unchanged under any conditions. However, tyrosine and all branched chain amino acids and aromatic amino acids were sensitive to storage temperature and freeze-thaw.

Notably, the increased levels of aspartate and glutamate were observed during 4°C and 22°C for extended storage periods in our study. This may be explained by asparagine and glutamine being converted to their dicarboxylic acid counterparts by deamidation (Wright, 1991). Protein degradation is the key factor for the increase of amino acid concentration, especially for those amino acids occurring with high frequency in proteins, e.g. isoleucine and glycine. Tryptophan and phenylalanine increased distinctly after repeated freeze-thaw cycles given the protein degradation during thawing and refreezing. This assumption was supported by previous studies (Anton et al., 2015; Wood et al., 2008). Increased cystine could be explained by rapid oxidation from unstable cysteine to cystine at room temperature. However, because cystine levels also decreased during repeated freeze-thaw cycles, the reduction of both cysteine and cystine could be a result of oxidative conversion to unidentified derivates as previously described (Moriya et al., 2016). Up to four freeze-thaw cycles will not affect the stability of the metabolites which were inferred by other researchers (Anton et al., 2015). Conversely, our results indicated the freeze-thaw had a great effect on the stability of amino acids in biological samples.

### Conclusions

We investigated the stability of amino acids in serum samples which underwent prolonged storage at 4°C and 22°C, and repeated freeze-thaw cycles at −80°C using stable isotope iTRAQ labeling and liquid chromatography tandem mass spectrometry. The results indicated that significant changes to amino acid concentrations had occurred during the different preprocessing and pre-storage conditions. Optimization of serum samples will require precise control over the collection, preservation, and pretreatment of those serum samples. Standardization should also be pursued, for example, biological samples should avoid freeze-thaw before analysis. Furthermore, pretreatment should be shortened to minimize potential concentration changes of biomarkers and metabolites. This study provided a standard method for collection, preparation, transportation, and storage of serum samples throughout the quantitative analysis process for amino acids and metabonomics using liquid chromatography-mass spectrometry.

## MATERIALS AND METHODS

### Chemicals and reagents

The derivatization of 44 amino acids and related amines were conducted on the iTRAQ^®^ Reagent Kit 200 Assay (P/N: A1116, AB Sciex, USA), including phosphoserine (PSer), phosphoethanolamine (PEtN), taurine (Tau), asparagine (Asn), serine (Ser), hydroxyproline (Hyp), glycine (Gly), glutamine (Gln), aspartate (Asp), ethanolamine (EtN), histidine (His), threonine (Thr), citrulline (Cit), sarcosine (Sar), *β*-alanine (*β*-Ala), alanine (Ala), glutamate (Glu), 1-methylhistidine, (1MHis), 3-methylhistidine (3MHis), argininosuccinic acid (Asa), carnosine (Car), homocitrulline (Hcit), arginine (Arg), *α*-aminoadipic acid (Aad), *γ*-aminobutyric acid (GABA), *β*-aminoisobutyric acid (*β*-Aib), *α*-amino-N-butyric acid (*α*-Abu), anserine (Ans), *δ*-hydroxylysine (*δ*-Hyl), proline (Pro), ornithine (Orn), cystathionine (Cth), cystine (Cys), lysine (Lys), methionine (Met), valine (Val), norvaline (Nva), tyrosine (Tyr), homocysteine (Hcy), isoleucine (Ile), leucine (Leu), norleucine (Nle), phenylalanine (Phe), and tryptophan (Trp). Heptafluorobutyric acid (≥99.5%) was obtained from Sigma-Aldrich (Switzerland) for mobile phase preparation. Acetonitrile and formic acid were purchased from Merck (Darmstadt, Germany). All chemicals and reagents were of appropriate analytical grades.

### Serum sample preparation under different storage conditions

The blood samples were collected from 11 healthy volunteers (six male, five female, aged 18–40 years) after overnight fasting in Beijing Chao-Yang Hospital in September 2014. All participants provided written informed consent. The study was carried out under the approval of the Ethics Committee of the Beijing Chao-Yang Hospital affiliated with the Beijing Capital Medical University and all experiments were performed in accordance with the relevant guidelines and regulations. The blood sample collection was completed by professional medical staff. Patient information was verified, and the collection time was recorded. Approximately 4 ml of venous whole blood was collected from the cubital fossa vein of the participants' upper limb. The collected blood samples were placed in non-anticoagulant tubes and allowed to coagulate at 4°C for 10 min, followed by centrifugation at 3500 rpm at 4°C for 10 min. The supernatant was separated by vortexing and centrifugation at the conditions described above. Three ml of serum was split into 200 μl aliquots.

**Fig. 6.**
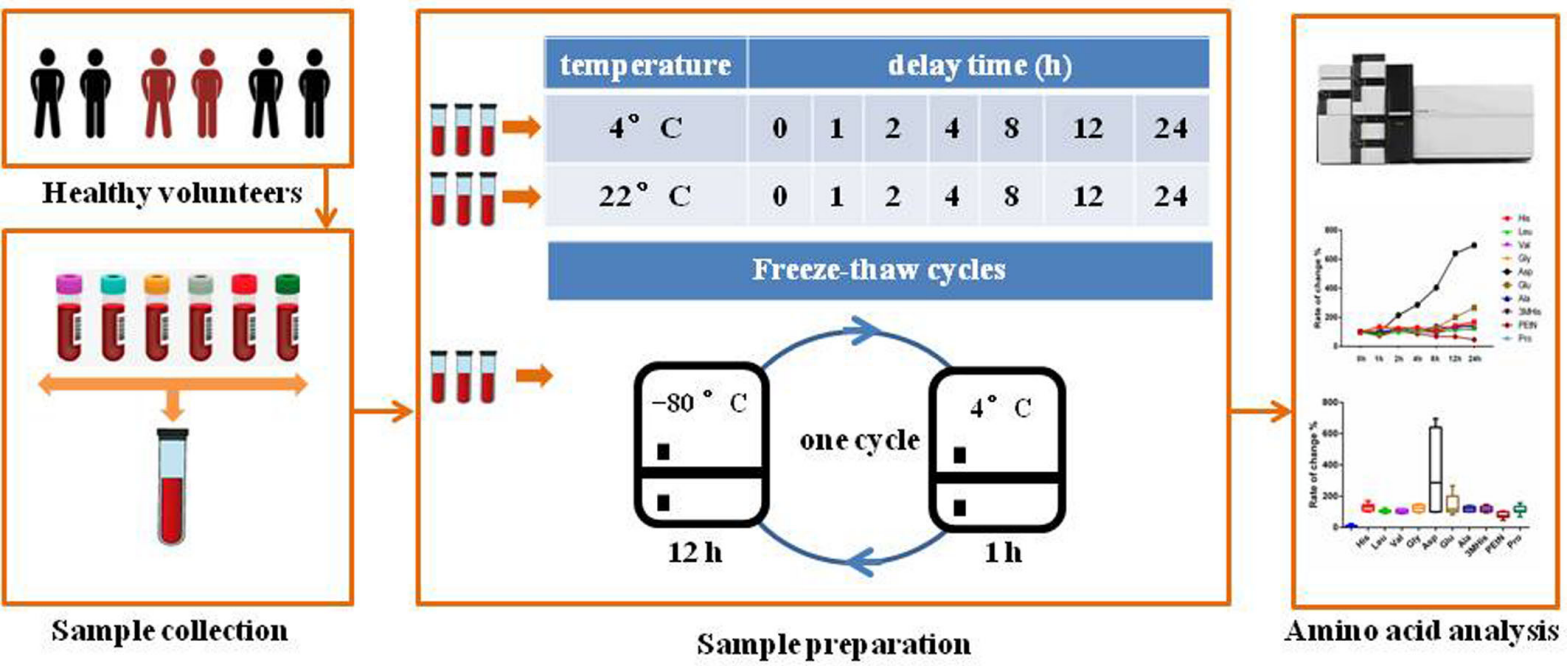
**Overview of stability investigation of amino acids and related amines in human serum under three conditions of preprocessing and pre-storage.** (1) The serum specimens placed at 4°C for 0, 1, 2, 4, 8, 12 and 24 h; (2) samples were placed at 22°C for 0, 1, 2, 4, 8, 12 and 24 h; (3) serum aliquots were stored at −80°C and subjected to up to three freeze-thaw cycles. A freeze-thaw cycle consisted of removing aliquots from −80°C, thawing aliquots for 1 h at 4°C, and setting them back to −80°C for 12 h.

Each of the initial 200 μl of serum were subjected to three handling protocols separately: (1) the serum specimens were placed at 4°C for 0, 1, 2, 4, 8, 12 and 24 h; (2) the samples were stored at 22°C for 0, 1, 2, 4, 8, 12 and 24 h; (3) the serum aliquots were stored at −80°C and subjected to three freeze-thaw cycles. A freeze-thaw cycle consisted of removing aliquots from −80°C, thawing aliquots for 1 h at 4°C, and setting them back to −80°C for 12 h. After the given time interval, 40 μl of serum was taken to be analyzed. At each time point and set of conditions, four parallel 40 μl samples of serum were analyzed. The experimental design for investigating amino acid and related amine stability in human serum under different storage conditions is shown in Fig. 6. Serum specimens were immediately frozen in liquid nitrogen.

### Targeted metabolite quantification

**Fig. 7.**
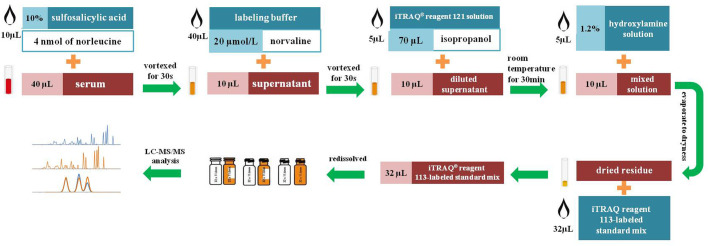
**Workflow indicating the procedures for biological sample preparation and amino acid derivatization using ITRAQ reagents.** The norleucine and norvaline were applied for the evaluation of extraction and derivatization efficiency. The iTRAQ-113-labeled amino acids were used as the isotopic ISs for the normalization of their corresponding iTRAQ-121-labeled amino acids in biological samples. The hydroxylamine solution was applied for the termination of derivatization reaction.

Each sample was measured according to our previously reported method (An et al., 2020). Quadruplicate samples were processed for analysis and tested in parallel at the same temperature and condition for equal duration. A workflow indicating the procedure for biological sample preparation and amino acid derivatization using ITRAQ^®^ reagents is shown in Fig. 7. An aliquot of 40 μl of serum samples was supplemented with 10 μl of 10% sulfosalicylic acid containing 4 nmol of norleucine. The norleucine was applied as an internal standard (IS) for the evaluation of extraction efficiency. The mixture underwent vortexing for 30 s and was centrifuged at 10,000* **g*** for 2 min at 4°C. 40 μL of labeling buffer (containing 20 μmol/l norvaline for evaluation of derivatization efficiency) was added to 10 μl of supernatant. Subsequently, 10 μl of the diluted supernatant was mixed with 5 μl iTRAQ^®^ reagent 121 solution (diluted with 70 μl isopropanol) followed by vortexing for 30 s. The derivatization reaction was terminated by adding 5 μl of 1.2% hydroxylamine solution followed by incubating at room temperature for 30 min. The resulting mixture was evaporated to dryness in an atmosphere of nitrogen. Finally, we re-dissolved the dried residue with 32 μl iTRAQ^®^ reagent 113-labeled standard mix (An et al., 2020).

### Instrumental analysis

The analysis of amino acid derivatives was performed on a Shimadzu LC-20AT liquid chromatography system couple with an API 3200 Qtrap™ mass spectrometer. Chromatography was performed on an XBridge Shield RP18 column (5 μm, 150 mm×4.6 mm) where the column temperature was held constant at 50°C with an injection volume of 3 μl. Mobile phase A was water and phase B was acetonitrile, both containing 0.01% heptafluorobutyric acid and 0.1% formic acid at a flowrate of 0.8 ml/min. The separation was conducted under the following gradient: 0–11 min, 0–20% B; 11–11.5 min, 20–100% B; 11.5–14 min, 100% B; 14–14.1 min, 100–0% B; 14.1–21 min, 0% B. Multiple reaction monitoring (MRM) in positive ionization mode was used for amino acid derivatives detection. Parameters including ESI voltage, entrance potential (EP), and declustering potential (DP) were set up to +5.5 kV, 30 V and 10 V, respectively. Furthermore, a 540°C source temperature, 20 psi curtain gas flow, 50 psi nebulizer gas flow, 60 psi source gas flow, and 30 eV collision energy (CE) were used in this study. Data acquisition was carried out by Analyst 1.5.1 software (Applied Biosystems Sciex).

### Data processing and statistical analysis

Peak integration of iTRAQ-121 and iTRAQ-113 labeled amino acids was carried out by the Analyst 1.5.1 software. The 113-labeled IS and 121-labeled amino acids are a pair of isotopic compounds with the same mass that elute at the same retention time in this iTRAQ-based strategy. The iTRAQ-113-labeled amino acids were used as the isotopic ISs to normalize their corresponding iTRAQ-121-labeled amino acids. This allows for the quantification of each amino acid according to their actual abundance in the biological samples. The obtained amino acid quantitative data were further used for statistical analysis. SIMICA-P+ 13.0 (Umetrics AB, Umeå, Sweden) was employed for PCA analysis. The amino acid quantitative results at different times were compared with the amino acid concentration at 0 h to identify the extent of any change, the percentage difference between the timepoint results and the 0 h result is referred to as the change ratio. The changes to amino acid levels that were affected by various environmental factors were identified by using a *t*-test (*P*<0.05).

## Supplementary Material

Supplementary information
