# Supplementary material for: Stability of amino acids and related amines in human serum under different preprocessing and pre-storage conditions based on iTRAQ®-LC-MS/MS
Source: Biol Open. 2021 Feb 26;10(2):bio055020. doi: 10.1242/bio.055020 (PMC7928226; doi:10.1242/bio.055020)
Supplement: Supplementary information [file biolopen-10-055020-s1.pdf]

Table S1 The concentration and change ratio of 17 amino acids which changed significantly ($p<0.05$) after serum specimens incubated at 4 °C for 24h.

	Analyte (μmol/L)																
**Temperture and delay time**	**His**	**Leu**	**Lys**	**Phe**	**Trp**	**Val**	**Gly**	**Ser**	**Tau**	**Asp**	**Glu**	**Cys**	**Ala**	**bAla**	**3MHis**	**PEtN**	**Pro**
0h-1	61.02	80.82	108.65	54.64	30.33	160.47	236.42	145.47	36.09	2.24	7.45	112.17	216.87	11.88	1.85	1.29	117.91
0h-2	57.85	88.25	101.16	51.86	34.72	163.05	237.33	140.03	38.34	1.34	9.71	78.64	299.41	10.57	2.30	1.53	108.97
0h-3	46.26	103.85	92.96	62.05	39.45	181.50	295.67	187.40	52.97	0.68	14.01	79.71	343.24	15.52	2.61	1.79	96.49
0h-4	31.91	96.34	106.79	55.82	36.46	166.28	200.44	157.07	41.78	2.16	9.55	115.24	276.87	10.01	2.06	1.77	124.27
mean	49.26	92.31	102.39	56.09	35.24	167.82	242.47	157.49	42.29	1.60	10.18	96.44	284.10	12.00	2.20	1.59	111.91
SD	13.19	9.96	7.05	4.31	3.81	9.42	39.41	21.17	7.49	0.74	2.76	19.98	52.61	2.47	0.32	0.24	12.04
4°C-1h-1	75.78	73.27	116.15	50.00	36.20	141.02	290.14	187.76	52.39	0.75	7.60	76.29	255.77	2.19	3.44	1.45	84.74
4°C-1h-2	74.43	91.29	130.33	54.01	44.53	142.86	302.63	194.04	64.58	2.72	7.33	70.35	260.08	3.41	0.00	1.09	64.92
4°C-1h-3	52.81	87.30	171.72	53.51	40.30	152.14	323.08	159.37	63.01	1.63	11.52	57.46	342.91	3.43	1.68	0.87	85.95
4°C-1h-4	65.88	77.92	145.25	54.37	37.12	159.51	282.61	132.67	55.61	1.27	6.92	59.51	293.22	4.40	1.11	1.39	73.61
mean	67.23	82.44	140.86	52.97	39.54	148.88	299.61	168.46	58.90	1.59	8.34	65.90	287.99	3.36	2.08	1.20	77.30
SD	10.56	8.30	23.76	2.01	3.76	8.60	17.69	28.23	5.84	0.83	2.14	8.94	40.25	0.90	1.44	0.27	9.95
T-test	0.05	0.14	0.08	0.26	0.15	0.03	0.02	0.65	0.02	0.99	0.07	0.06	0.83	0.01	0.48	0.17	0.03
**change ratio (%)**	**136%**	**89%**	**138%**	**94%**	**112%**	**89%**	**124%**	**107%**	**139%**	**99%**	**82%**	**68%**	**101%**	**28%**	**94%**	**75%**	**69%**
4°C-2h-1	71.22	74.72	95.66	44.48	32.15	160.17	247.01	130.19	34.27	3.26	10.15	81.33	265.86	7.50	2.80	1.47	108.77
4°C-2h-2	76.53	85.19	110.45	48.90	32.06	161.86	336.76	130.21	41.16	3.74	10.68	75.31	292.72	11.03	3.24	1.47	147.30
4°C-2h-3	35.97	86.59	119.32	54.06	35.92	134.26	323.99	125.14	47.44	2.58	8.32	74.89	330.49	3.07	1.59	1.54	82.88
4°C-2h-4	57.25	115.55	160.42	62.50	41.53	213.97	361.37	194.75	50.75	4.12	18.46	125.96	458.15	13.76	0.00	2.16	140.86
mean	60.24	90.51	121.46	52.48	35.41	167.56	317.28	145.07	43.40	3.43	11.90	89.37	336.80	8.84	2.55	1.66	119.95
SD	18.11	17.51	27.74	7.74	4.46	33.42	49.35	33.21	7.27	0.66	4.49	24.57	85.13	4.62	1.45	0.33	29.91
T-test	0.25	0.83	0.27	0.41	0.94	0.99	0.12	0.59	0.75	0.01	0.61	0.47	0.33	0.44	0.72	0.66	0.55
**change ratio (%)**	**122%**	**98%**	**119%**	**94%**	**100%**	**100%**	**131%**	**92%**	**103%**	**214%**	**117%**	**93%**	**119%**	**74%**	**116%**	**104%**	**107%**

4°C-4h-1	65.80	78.19	120.38	45.90	28.57	129.41	246.65	124.79	36.72	3.07	12.60	62.30	299.31	13.99	1.53	1.62	81.63
4°C-4h-2	46.35	90.93	136.74	57.50	42.40	184.39	285.31	109.98	45.38	6.58	10.75	97.26	358.29	12.59	2.66	1.23	173.30
4°C-4h-3	67.81	97.15	105.77	53.44	33.31	160.26	294.43	172.18	46.82	4.04	10.19	64.86	417.76	12.24	2.85	1.62	108.62
4°C-4h-4	75.35	107.03	135.68	61.02	39.69	212.86	363.39	149.62	44.98	4.63	12.69	163.83	404.64	13.99	2.87	1.06	93.69
mean	63.83	93.32	124.64	54.46	35.99	171.73	297.45	139.14	43.47	4.58	11.56	97.06	370.00	13.20	2.48	1.38	114.31
SD	12.36	12.07	14.64	6.50	6.25	35.47	48.60	27.43	4.57	1.48	1.27	47.27	53.59	0.92	0.64	0.28	40.85
T-test	0.30	0.81	0.03	0.72	0.82	0.84	0.24	0.03	0.70	0.05	0.53	0.98	0.01	0.50	0.33	0.40	0.92
**change ratio (%)**	**130%**	**101%**	**122%**	**97%**	**102%**	**102%**	**123%**	**88%**	**103%**	**286%**	**114%**	**101%**	**130%**	**110%**	**112%**	**87%**	**102%**
4°C-8h-1	47.71	86.44	104.07	55.62	32.18	156.82	179.13	133.26	43.59	6.74	13.26	89.86	222.40	13.05	3.16	0.96	135.75
4°C-8h-2	42.22	88.95	130.04	47.45	36.07	173.81	254.51	111.25	33.43	5.54	12.84	60.63	234.01	17.31	1.86	1.20	109.65
4°C-8h-3	59.04	101.98	123.23	55.05	33.23	142.54	249.41	123.63	44.40	5.89	11.14	61.15	402.51	11.87	3.09	1.09	144.71
4°C-8h-4	61.67	99.05	130.34	60.49	32.76	149.00	229.41	169.59	42.58	7.81	15.20	86.86	314.57	11.33	3.83	1.17	135.45
mean	52.66	94.11	121.92	54.65	33.56	155.54	228.11	134.43	41.00	6.50	13.11	74.62	293.37	13.39	2.98	1.10	131.39
SD	9.23	7.57	12.35	5.39	1.73	13.50	34.41	25.11	5.10	1.01	1.67	15.91	83.51	2.71	0.82	0.11	15.12
T-test	0.78	0.34	0.10	0.62	0.46	0.33	0.56	0.25	0.74	0.00	0.24	0.00	0.76	0.56	0.21	0.01	0.15
**change ratio (%)**	**107%**	**102%**	**119%**	**97%**	**95%**	**93%**	**94%**	**85%**	**97%**	**405%**	**129%**	**77%**	**103%**	**112%**	**135%**	**69%**	**117%**
4°C-12h-1	85.78	98.95	80.54	66.52	38.09	180.57	282.61	193.84	41.18	10.94	20.91	99.06	495.80	11.18	2.01	1.24	162.52
4°C-12h-2	60.78	101.02	169.23	70.98	44.27	176.26	349.61	257.40	47.79	11.45	23.38	69.08	316.25	19.59	2.90	1.00	125.74
4°C-12h-3	73.89	97.04	203.76	58.64	31.78	206.02	306.36	176.70	54.19	9.58	19.01	107.41	281.11	13.35	2.26	1.12	172.17
4°C-12h-4	63.40	108.90	110.00	68.42	39.14	214.29	464.29	166.93	44.61	9.16	18.28	71.92	443.96	16.31	4.19	0.89	138.89
mean	70.96	101.48	140.88	66.14	38.32	194.28	350.72	198.72	46.94	10.28	20.39	86.87	384.28	15.11	2.84	1.06	149.83
SD	11.39	5.21	55.83	5.32	5.13	18.71	80.63	40.67	5.54	1.09	2.28	19.24	102.09	3.65	0.98	0.15	21.29
T-test	0.04	0.19	0.31	0.13	0.48	0.04	0.15	0.24	0.08	0.00	0.02	0.56	0.28	0.33	0.32	0.06	0.08
**change ratio (%)**	**144%**	**110%**	**138%**	**118%**	**109%**	**116%**	**145%**	**126%**	**111%**	**641%**	**200%**	**90%**	**135%**	**126%**	**129%**	**67%**	**134%**
4°C-24h-1	92.06	108.00	149.52	62.22	40.20	203.95	339.24	200.80	48.57	7.40	26.35	96.04	401.99	8.15	2.64	0.70	197.16
4°C-24h-2	94.55	118.46	124.90	73.80	42.86	188.68	339.29	128.90	49.77	17.23	26.33	135.08	401.34	8.55	4.07	0.65	162.66
4°C-24h-3	84.04	106.33	101.27	71.66	45.08	189.33	402.82	137.41	51.81	8.40	28.31	114.23	455.82	11.35	3.14	0.78	151.06

4°C-24h-4	66.82	125.81	186.39	65.16	45.82	202.08	364.53	152.49	45.35	11.61	27.38	68.43	319.80	13.19	2.97	0.79	193.55
mean	84.37	114.65	140.52	68.21	43.49	196.01	361.47	154.90	48.88	11.16	27.09	103.45	394.74	10.31	3.21	0.73	176.11
SD	12.53	9.17	36.38	5.42	2.53	8.13	30.03	32.12	2.70	4.43	0.95	28.27	56.11	2.39	0.62	0.07	22.77
T-test	0.00	0.04	0.09	0.04	0.00	0.04	0.00	0.91	0.14	0.02	0.00	0.79	0.03	0.39	0.03	0.00	0.00
**change ratio (%)**	**171%**	**124%**	**137%**	**122%**	**123%**	**117%**	**149%**	**98%**	**116%**	**696%**	**266%**	**107%**	**139%**	**86%**	**146%**	**46%**	**157%**

Biology Open • Supplementary information

Table S2 The concentration and change ratio of 18 amino acids which changed significantly (p<0.05) after serum specimens incubated at 22 °C for 24h.

	**Analyte (µmol/L)**																	
**Temperture and delay time**	**His**	**Ile**	**Leu**	**Lys**	**Met**	**Phe**	**Thr**	**Trp**	**Val**	**Asn**	**Asp**	**Glu**	**Gln**	**Orn**	**Cys**	**Ala**	**3MHis**	**PEtN**
0h-1	61.02	35.81	80.82	108.65	29.22	54.64	182.80	30.33	160.47	62.26	2.24	7.45	574.30	33.42	112.17	216.87	1.85	1.29
0h-2	57.85	41.93	88.25	101.16	28.00	51.86	164.22	34.72	163.05	38.83	1.34	9.71	669.98	31.54	78.64	299.41	2.30	1.53
0h-3	46.26	49.81	103.85	92.96	31.79	62.05	187.42	39.45	181.50	66.36	0.68	14.01	710.53	36.86	79.71	343.24	2.61	1.79
0h-4	31.91	42.17	96.34	106.79	29.60	55.82	105.05	36.46	166.28	57.14	2.16	9.55	539.71	32.82	115.24	276.87	2.06	1.77
mean	49.26	42.43	92.31	102.39	29.65	56.09	159.87	35.24	167.82	56.15	1.60	10.18	623.63	33.66	96.44	284.10	2.20	1.59
SD	13.19	5.73	9.96	7.05	1.58	4.31	37.90	3.81	9.42	12.14	0.74	2.76	79.95	2.27	19.98	52.61	0.32	0.24
22°C-1h-1	84.51	49.53	113.20	120.97	30.20	59.07	156.89	33.04	169.03	68.15	4.99	12.30	467.53	19.93	79.48	261.56	3.27	1.78
22°C-1h-2	84.87	41.46	83.64	139.82	23.87	51.49	92.40	28.58	182.96	48.02	3.30	11.48	583.33	29.69	74.56	324.72	2.20	0.99
22°C-1h-3	43.38	43.46	102.04	124.53	31.76	59.93	148.30	42.63	185.62	75.46	4.58	13.09	403.51	37.43	78.53	239.60	2.42	1.29
22°C-1h-4	42.81	52.95	108.95	123.66	36.58	69.70	130.93	45.91	212.67	59.59	5.33	15.99	542.97	53.07	148.27	389.16	3.26	1.45
mean	63.89	46.85	101.96	127.24	30.60	60.05	132.13	37.54	187.57	62.80	4.55	13.22	499.34	35.03	95.21	303.76	2.79	1.38
SD	24.02	5.32	13.05	8.52	5.25	7.47	28.60	8.09	18.25	11.80	0.89	1.96	79.90	14.00	35.44	67.41	0.56	0.33
T-test	0.12	0.42	0.34	0.03	0.71	0.35	0.27	0.52	0.13	0.03	0.01	0.16	0.15	0.86	0.93	0.69	0.26	0.43
**change ratio (%)**	**130%**	**110%**	**110%**	**124%**	**103%**	**107%**	**83%**	**107%**	**112%**	**112%**	**284%**	**130%**	**80%**	**104%**	**99%**	**107%**	**127%**	**86%**
22°C-2h-1	58.87	47.16	117.81	179.17	39.87	67.36	147.89	46.70	169.18	40.73	6.15	17.19	811.48	27.39	124.68	449.90	1.98	1.41
22°C-2h-2	67.68	45.19	86.73	112.12	33.61	50.97	219.86	38.16	182.42	50.42	5.46	12.54	1020.62	45.18	73.27	348.84	3.95	1.57
22°C-2h-3	57.54	52.94	99.59	101.09	37.42	57.85	97.38	36.75	183.55	48.58	3.64	19.92	563.47	38.80	97.30	277.61	2.96	1.61
22°C-2h-4	93.40	51.67	106.77	148.21	31.68	59.39	175.32	40.10	200.79	70.95	4.15	14.48	681.72	54.55	122.26	244.70	3.49	1.36
mean	69.37	49.24	102.73	135.15	35.64	58.89	160.11	40.43	183.99	52.67	4.85	16.03	769.32	41.48	104.38	330.26	3.09	1.49
SD	16.64	3.67	13.03	35.58	3.69	6.73	51.27	4.40	12.97	12.89	1.16	3.22	195.77	11.40	24.15	90.83	0.85	0.12
T-test	0.25	0.05	0.35	0.11	0.04	0.50	1.00	0.29	0.11	0.74	0.01	0.03	0.26	0.29	0.21	0.54	0.10	0.44
**change ratio (%)**	**141%**	**116%**	**111%**	**132%**	**120%**	**105%**	**100%**	**115%**	**110%**	**94%**	**302%**	**158%**	**123%**	**123%**	**108%**	**116%**	**140%**	**93%**
22°C-4h-1	108.38	49.08	111.76	134.34	29.45	72.13	209.70	37.64	197.53	34.87	11.44	20.82	625.32	33.50	59.07	411.42	2.36	1.04

Biology Open • Supplementary information

22°C-4h-2	95.65	55.10	131.13	120.22	28.38	65.53	206.28	37.24	177.00	61.28	10.43	13.20	742.39	71.84	103.08	389.51	1.94	1.10
22°C-4h-3	79.08	56.42	109.30	149.53	29.62	69.91	191.63	42.84	187.17	52.65	12.27	18.29	553.81	30.70	51.39	389.58	3.07	1.02
22°C-4h-4	73.52	48.59	118.24	135.79	31.75	59.45	154.98	35.79	202.65	58.84	11.68	30.60	811.56	45.83	110.18	404.34	2.56	0.70
mean	89.16	52.30	117.61	134.97	29.80	66.75	190.65	38.38	191.09	51.91	11.46	20.73	683.27	45.47	80.93	398.71	2.48	0.97
SD	15.89	4.04	9.77	11.98	1.41	5.59	25.04	3.08	11.39	11.93	0.77	7.30	115.57	18.77	29.98	10.97	0.47	0.18
T-test	0.00	0.01	0.05	0.03	0.88	0.04	0.06	0.15	0.06	0.72	0.00	0.08	0.54	0.33	0.42	0.04	0.28	0.04
**change ratio (%)**	**181%**	**123%**	**127%**	**132%**	**100%**	**119%**	**119%**	**109%**	**114%**	**92%**	**715%**	**204%**	**110%**	**135%**	**84%**	**140%**	**113%**	**61%**
22°C-8h-1	35.02	36.30	78.80	98.18	21.62	55.47	100.30	29.90	163.51	34.56	7.81	19.17	613.79	37.47	83.15	271.32	1.67	0.61
22°C-8h-2	49.34	35.86	82.92	84.52	23.58	59.60	151.74	32.50	156.99	39.49	8.18	19.90	430.26	36.76	51.00	288.40	2.68	0.61
22°C-8h-3	72.82	50.61	111.35	129.02	33.41	70.28	200.87	36.90	193.72	71.06	12.23	32.02	984.57	46.96	40.31	318.60	2.69	0.98
22°C-8h-4	78.46	42.77	96.69	126.55	23.49	53.97	171.34	33.98	195.80	52.26	11.26	20.63	603.38	39.84	70.90	176.82	4.08	0.60
mean	58.91	41.38	92.44	109.57	25.52	59.83	156.06	33.32	177.51	49.34	9.87	22.93	658.00	40.26	61.34	263.78	2.78	0.70
SD	20.31	6.92	14.75	21.79	5.33	7.36	42.31	2.92	20.12	16.29	2.21	6.09	233.42	4.66	19.29	61.18	0.99	0.19
T-test	0.60	0.58	0.97	0.61	0.13	0.23	0.91	0.03	0.29	0.42	0.01	0.01	0.77	0.02	0.00	0.57	0.33	0.00
**change ratio (%)**	**120%**	**98%**	**100%**	**107%**	**86%**	**107%**	**98%**	**95%**	**106%**	**88%**	**616%**	**225%**	**106%**	**120%**	**64%**	**93%**	**126%**	**44%**
22°C-12h-1	50.83	41.24	93.88	175.88	30.36	62.19	127.27	36.05	181.61	66.68	6.45	19.23	484.18	43.94	102.12	307.41	3.40	0.79
22°C-12h-2	51.79	47.80	111.33	151.81	30.74	67.16	131.38	33.85	195.28	39.13	13.16	29.19	556.23	42.48	82.28	327.01	3.81	0.58
22°C-12h-3	61.65	43.87	91.15	150.54	30.79	65.12	112.45	33.92	193.60	64.68	12.33	29.10	482.97	59.04	80.07	298.11	0.00	0.66
22°C-12h-4	23.60	38.33	83.10	121.12	31.88	54.24	90.43	27.50	149.32	37.34	11.15	15.33	591.13	40.37	111.04	277.36	1.28	0.62
mean	46.97	42.81	94.86	149.84	30.94	62.18	115.38	32.83	179.95	51.96	10.77	23.21	528.63	46.46	93.88	302.47	2.83	0.66
SD	16.33	4.02	11.89	22.41	0.65	5.67	18.51	3.70	21.31	15.89	3.00	7.03	53.94	8.52	15.14	20.62	1.80	0.09
T-test	0.73	0.91	0.80	0.03	0.22	0.19	0.04	0.50	0.33	0.49	0.01	0.02	0.20	0.03	0.45	0.56	0.94	0.01
**change ratio (%)**	**95%**	**101%**	**103%**	**146%**	**104%**	**111%**	**72%**	**93%**	**107%**	**93%**	**672%**	**228%**	**85%**	**138%**	**97%**	**106%**	**128%**	**42%**
22°C-24h-1	106.76	54.77	118.03	153.43	41.16	78.77	202.06	43.18	195.82	43.94	9.33	96.75	692.92	47.42	57.06	323.70	2.56	0.00
22°C-24h-2	86.60	51.23	99.43	140.42	32.50	63.42	117.22	38.60	195.85	36.90	8.93	28.24	693.25	45.91	43.64	311.45	3.46	0.16
22°C-24h-3	105.63	60.59	137.78	182.07	43.36	80.63	192.15	42.49	187.31	57.10	8.01	36.51	915.43	72.67	51.97	420.14	3.33	0.34
22°C-24h-4	95.35	51.79	118.78	188.19	42.03	72.01	133.05	34.54	210.30	58.83	14.41	26.60	676.13	72.42	30.56	530.76	3.03	0.00

mean	98.58	54.59	118.51	166.03	39.76	73.71	161.12	39.70	197.32	49.19	10.17	47.03	744.43	59.61	45.81	396.51	3.09	0.25
SD	9.50	4.29	15.66	22.83	4.92	7.79	42.25	3.99	9.54	10.55	2.88	33.43	114.28	14.95	11.57	101.85	0.40	0.16
T-test	0.01	0.01	0.02	0.02	0.01	0.01	0.95	0.24	0.04	0.21	0.01	0.13	0.05	0.03	0.03	0.12	0.00	0.00
**change ratio (%)**	**200%**	**129%**	**128%**	**162%**	**134%**	**131%**	**101%**	**113%**	**118%**	**88%**	**634%**	**462%**	**119%**	**177%**	**47%**	**140%**	**140%**	**16%**

Biology Open • Supplementary information

Table S3-1 The concentration and change ratio of 14 amino acids in the serum altered after three freeze-thaw cycles compared with the samples without freeze-thaw process ($p<0.05$). The freeze-thaw cycle including samples were brought from −80 °C, placed at 4 °C for one hour an delivered to −80 °C for 12 hours.

	Analyte (μmol/L)														
**sample style**	**Arg**	**His**	**Ile**	**Leu**	**Lys**	**Met**	**Phe**	**Glu**	**Gln**	**Thr**	**Trp**	**Val**	**Gly**	**Ser**	**Ala**
0h-sample-1	82.78	61.02	35.81	80.82	108.65	29.22	54.64	7.45	574.30	182.80	30.33	160.47	236.42	145.47	216.87
0h-sample-2	69.45	57.85	41.93	88.25	101.16	28.00	51.86	9.71	669.98	164.22	34.72	163.05	237.33	140.03	299.41
0h-sample-3	102.10	46.26	49.81	103.85	92.96	31.79	62.05	14.01	710.53	187.42	39.45	181.50	295.67	187.40	343.24
0h-sample-4	127.54	31.91	42.17	96.34	106.79	29.60	55.82	9.55	539.71	105.05	36.46	166.28	200.44	157.07	276.87
0h-sample-mean	95.47	49.26	42.43	92.31	102.39	29.65	56.09	10.18	623.63	159.87	35.24	167.82	242.47	157.49	284.10
SD	25.24	13.19	5.73	9.96	7.05	1.58	4.31	2.76	79.95	37.90	3.81	9.42	39.41	21.17	52.61
freeze-thaw cycles-sample-1	110.15	114.18	92.76	190.90	205.93	44.40	65.32	43.90	673.37	257.71	49.16	270.74	183.53	180.78	414.83
freeze-thaw cycles-sample-2	100.30	100.00	84.42	169.97	90.14	40.63	60.22	31.92	485.66	216.15	46.15	271.53	204.60	169.92	353.04
freeze-thaw cycles-sample-3	97.89	60.59	98.13	178.57	160.28	44.23	69.68	35.07	340.71	115.91	51.54	274.76	270.59	101.97	391.84
freeze-thaw cycles-sample-4	123.62	81.22	75.33	176.85	217.96	53.07	60.70	32.48	459.33	115.92	41.57	281.87	209.77	80.73	420.20
freeze-thaw cycles-sample-mean	107.99	89.00	87.66	179.07	168.58	45.58	63.98	35.84	489.77	176.42	47.11	274.72	217.12	133.35	394.98
SD	11.69	23.26	9.97	8.72	57.90	5.29	4.44	5.54	137.68	71.90	4.30	5.07	37.41	49.47	30.54
T-test	0.28	0.02	0.00	0.00	0.09	0.01	0.01	0.01	0.27	0.64	0.02	0.00	0.15	0.52	0.06
**change ratio (%)**	**13.12%**	**80.66%**	**106.60%**	**93.98%**	**64.64%**	**53.72%**	**14.06%**	**252.12%**	**-21.47%**	**10.35%**	**33.68%**	**63.70%**	**-10.45%**	**-15.33%**	**39.03%**

Table S3-2 The concentration and change ratio of 14 amino acids in the serum altered after three freeze-thaw cycles compared with the samples without freeze-thaw process ($p<0.05$). The freeze-thaw cycle including samples were brought from −80 °C, placed at 4 °C for one hour an delivered to −80 °C for 12 hours.

	Analyte (μmol/L)															
**sample style**	**Tau**	**Tyr**	**Asn**	**Cit**	**Orn**	**Cys**	**Abu**	**Pro**	**βAla**	**βAib**	**EtN**	**Hyp**	**1MHis**	**3MHis**	**PEtN**	**Asp**
0h-sample-1	36.09	43.12	62.26	18.14	33.42	112.17	9.01	117.91	11.88	1.66	11.24	7.34	1.83	1.85	1.29	2.24
0h-sample-2	38.34	42.94	38.83	45.92	31.54	78.64	15.90	108.97	10.57	0.60	8.77	6.86	1.81	2.30	1.53	1.34
0h-sample-3	52.97	52.68	66.36	71.23	36.86	79.71	20.45	96.49	15.52	0.92	12.97	6.67	1.25	2.61	1.79	0.68
0h-sample-4	41.78	58.51	57.14	17.86	32.82	115.24	11.68	124.27	10.01	1.65	16.15	6.23	2.36	2.06	1.77	2.16
0h-sample-mean	42.29	49.31	56.15	38.29	33.66	96.44	14.26	111.91	12.00	1.21	12.28	6.77	1.81	2.20	1.59	1.60
SD	7.49	7.64	12.14	25.61	2.27	19.98	5.01	12.04	2.47	0.53	3.10	0.46	0.46	0.32	0.24	0.74
freeze-thaw cycles-sample-1	63.37	61.69	53.38	25.19	50.00	65.42	27.87	116.52	5.71	1.49	14.02	7.07	0.00	2.03	2.04	0.00
freeze-thaw cycles-sample-2	71.52	69.18	41.80	16.37	42.76	50.98	18.30	144.63	3.26	2.26	11.40	8.99	0.00	1.50	1.69	0.00
freeze-thaw cycles-sample-3	64.92	61.43	69.23	26.20	55.91	49.08	22.62	98.73	3.83	3.28	11.26	8.64	0.00	1.88	1.83	0.00
freeze-thaw cycles-sample-4	55.05	66.97	48.85	9.02	46.82	56.06	37.36	110.15	2.49	2.82	18.17	6.45	0.00	0.00	1.44	0.00
freeze-thaw cycles-sample-mean	63.71	64.82	53.32	19.19	48.87	55.39	26.54	117.51	3.82	2.46	13.71	7.79	0.00	1.81	1.75	0.00
SD	6.77	3.87	11.63	8.10	5.55	7.31	8.21	19.52	1.38	0.77	3.23	1.22	0.00	0.93	0.25	0.00
T-test	0.03	0.04	0.46	0.19	0.00	0.01	0.13	0.63	0.01	0.10	0.27	0.20	0.00	0.16	0.53	0.02
**change ratio (%)**	50.65%	31.45%	-5.04%	-49.87%	45.20%	-42.57%	86.06%	5.00%	-68.14%	103.84%	11.65%	14.95%	-100%	-17.97%	9.84%	-100.0%
